# Peri-operative chemotherapy for resectable colorectal lung metastasis: a systematic review and meta-analysis

**DOI:** 10.1007/s00432-020-03142-9

**Published:** 2020-02-08

**Authors:** Yuting Li, You Qin

**Affiliations:** Cancer Center, Union Hospital, Tongji Medical College, Huazhong University of Science and Technology, Wuhan, 430022 People’s Republic of China

**Keywords:** Colorectal cancer, Pulmonary metastases, Chemotherapy, Meta-analysis

## Abstract

**Purpose:**

Several studies have evaluated surgical resection of pulmonary metastases as a standard treatment option for colorectal cancer (CRC) patients with resectable pulmonary metastases. However, the role of peri-operative chemotherapy after complete resection of pulmonary metastases from CRC patients is still controversial. This systematic review and meta-analysis is aimed to investigate the clinical efficacy of peri-operative chemotherapy after resection of CRC pulmonary metastases.

**Methods:**

PubMed, the Cochrane Library databases, and Embase were searched for studies evaluating the effect of peri-operative chemotherapy on the survival of patients with CRC after pulmonary metastasectomy. The hazard ratio (HR) was used for analyzing overall survival (OS) and progression-free survival (PFS)/recurrence-free survival (RFS)/disease-free survival (DFS).

**Results:**

Eight studies were included in the final analysis. The outcome showed that peri-operative chemotherapy had a significant favourable effect on OS (HR 0.83, 95% CI 0.75–0.92, *p* < 0.05) and PFS/RFS/DFS (HR 0.67, 95% CI 0.53–0.86, *p* < 0.05) in patients who received pulmonary metastasectomy. Multivariate analysis also validated this result (OS: HR 0.56, 95% CI 0.36–0.86, *p* < 0.05; PFS/RFS/DFS: HR 0.64, 95% CI 0.46–0.87, *p* < 0.05). There was a significant benefit in peri-operative group on OS and PFS/RFS/DFS in studies with R0 resection of pulmonary metastases (OS: HR 0.72, 95% CI 0.53–0.97, *p* < 0.05; PFS/RFS/DFS: HR 0.72, 95% CI 0.54–0.95, *p* < 0.05) and metachronous pulmonary metastases (OS: HR 0.40, 95% CI 0.22–0.75, *p* < 0.05; PFS/RFS/DFS: HR 0.67, 95% CI 0.49–0.92, *p* < 0.05).

**Conclusion:**

Our meta-analysis demonstrated a significant difference in favor of peri-operative chemotherapy in CRC patients who underwent resection of pulmonary metastases. More clinical data and studies are needed to validate the findings of our study.

**Electronic supplementary material:**

The online version of this article (10.1007/s00432-020-03142-9) contains supplementary material, which is available to authorized users.

## Background

CRC is the third most common cancer in the world. It was estimated that more than one million cases and 600 thousands deaths occurred in 2012 (Torre et al. 2015). The lung is known to be the second most frequent site of metastases. 19% of CRC patients were found to have synchronous metastases (present at the time of CRC diagnosis) before treatment, 11% of whom had lung metastases (Mitry et al. 2010). About 2–7% of patients have isolated metastases, while about 10% of patients will have synchronous pulmonary and liver metastases (Johnston et al. 2012).

Despite the lack of randomized studies, pulmonary metastasection has been recognized as a standard treatment for CRC patients with resectable pulmonary metastases. A pooled analysis of CRC pulmonary oligometastasis found that 5-year survival rate after pulmonary metastasectomy was 54.3% (Salah et al. 2012). In accordance with this finding, a series of studies demonstrated an overall 5-year survival rate of approximately 30.5–54.4% after resection of pulmonary metastases (Goya et al. 1989; McAfee et al. 1992; McCormack et al. 1992; Zampino et al. 2014; Salah et al. 2015). The prognostic factors of these patients included the number of pulmonary metastases, serum carcinoembryonic antigen (CEA), mediastinal and hilar lymph node involvement, and the disease-free interval (DFI) between the resection of the primary tumor and pulmonary metastases (Pfannschmidt et al. 2007).

Although surgical resection significantly improves the survival of these patients, relapses are still a challenge for treatment. It has been reported that the rate of relapse after resection of colorectal metastases ranges from 20 to 68% (Welter et al. 2007; Pfannschmidt et al. 2010; Guerrera et al. 2016). Therefore, it is still an urgent problem to find effective therapeutic strategies to reduce the risk of relapse in these patients (Brandi et al. 2016). However, the role of systemic chemotherapy in these patients is still inconsistent. Only a few studies have reported that peri-operative chemotherapy contributes to survival after resection of CRC pulmonary metastases(Muñoz Llarena et al. 2007; Kaira et al. 2011), while other studies have showed that these patients can’t benefit from systemic chemotherapy (Brandi et al. 2013; Park et al. 2017). A literature review of studies that examined whether peri-operative chemotherapy provided benefit for CRC patients with resection of lung metastases identified six retrospective studies (Guerrera et al. 2017). Five of these studies showed that adjuvant chemotherapy did not benefit OS, while a single study showed that chemotherapy positively impacted OS. However, meta-analysis was not conducted in this review. Therefore, we performed a systematic review and meta-analysis to evaluate the effectiveness of peri-operative chemotherapy in the treatment of CRC patients receiving resection of pulmonary metastases.

## Method

### Search strategy

Studies were identified by searching PubMed, Cochrane Library databases, and Embase without language restriction. The systematic review was undertaken on January 21, 2018. The following terms and keywords were used: pulmonary metastasis, pulmonary metastases, lung metastases, lung metastasis, colorectal cancer, colorectal neoplasms, colorectal tumor, colorectal carcinoma, rectal cancer, colon cancer, adjuvant chemotherapy, post-operative chemotherapy, neo-adjuvant chemotherapy, and peri-operative chemotherapy. Relevant articles and abstracts were reviewed and selected based on the defined inclusion and exclusion criteria.

### Inclusion criteria

Articles were included if they were published reports that included patients treated with or without peri-operative chemotherapy with resectable colorectal lung metastases. Only studies that reported patient survival data were included. Patients with synchronous or metachronous lung metastasis (develop during treatment or follow-up) were included.

### Exclusion criteria

Studies that did not examine long-term follow-up were excluded.

### Validity assessment

The quality of the selected studies was evaluated using the Newcastle–Ottawa Scale (NOS).

### Data extraction

The studies were independently examined by two investigators. First author, year of publication, study type, study population characteristics, number of subjects, length of follow-up, and outcomes were recorded. Publication bias was investigated by funnel plots.

### Statistical analysis

Stata software was used to evaluate the overall effect of peri-operative chemotherapy on survival. The results were obtained as the hazard ratio of PFS/RFS/DFS and OS. I^2^ was used to evaluate heterogeneity across studies. A *p* value of < 0.05 was considered statistically significant.

## Results

To date, there have been no reports of randomized trials that compared surgery to neo-adjuvant or adjuvant chemotherapy combined with surgery.

**Fig. 1:**
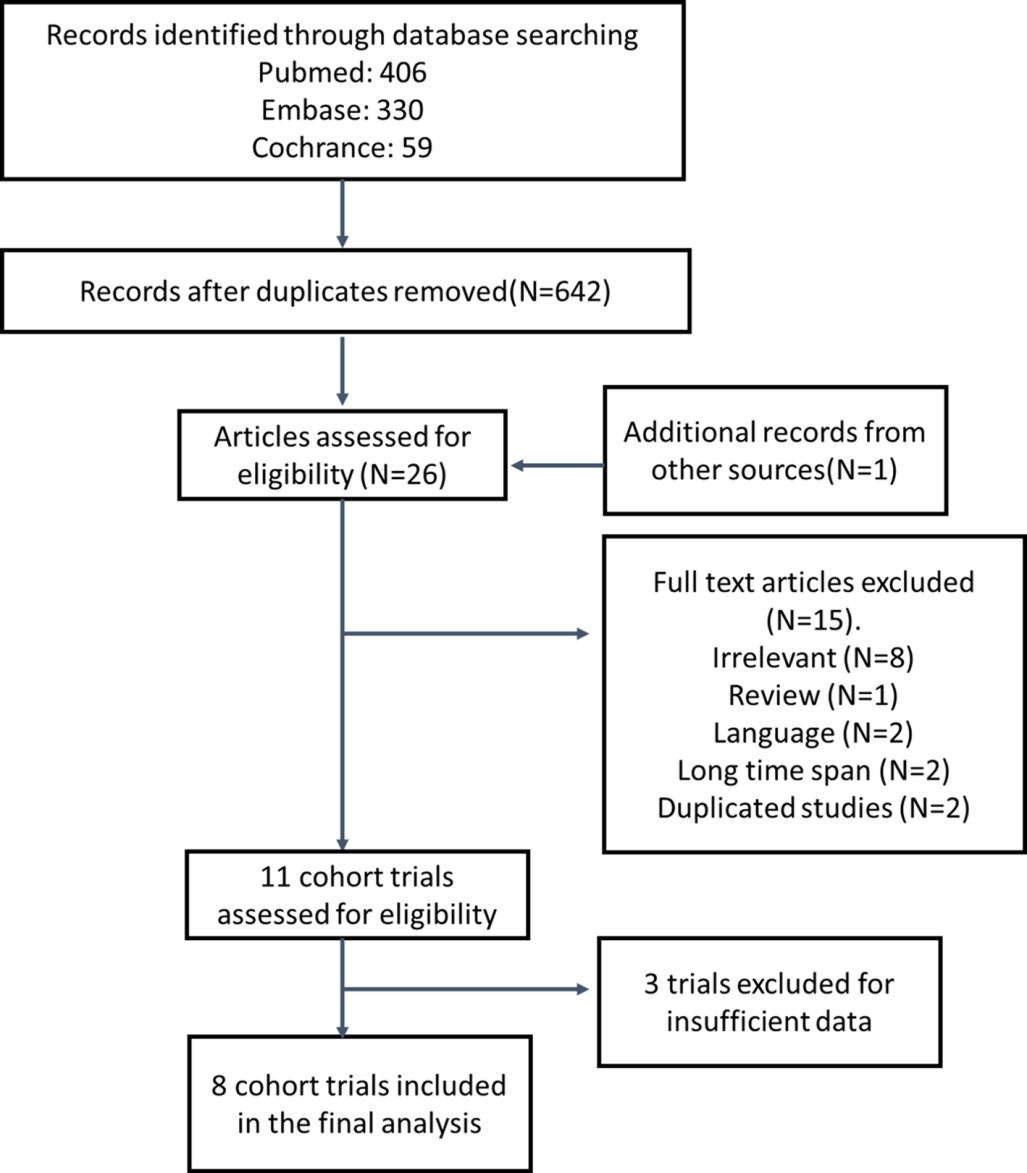
Flow chart of trial selection

**Fig. 2:**
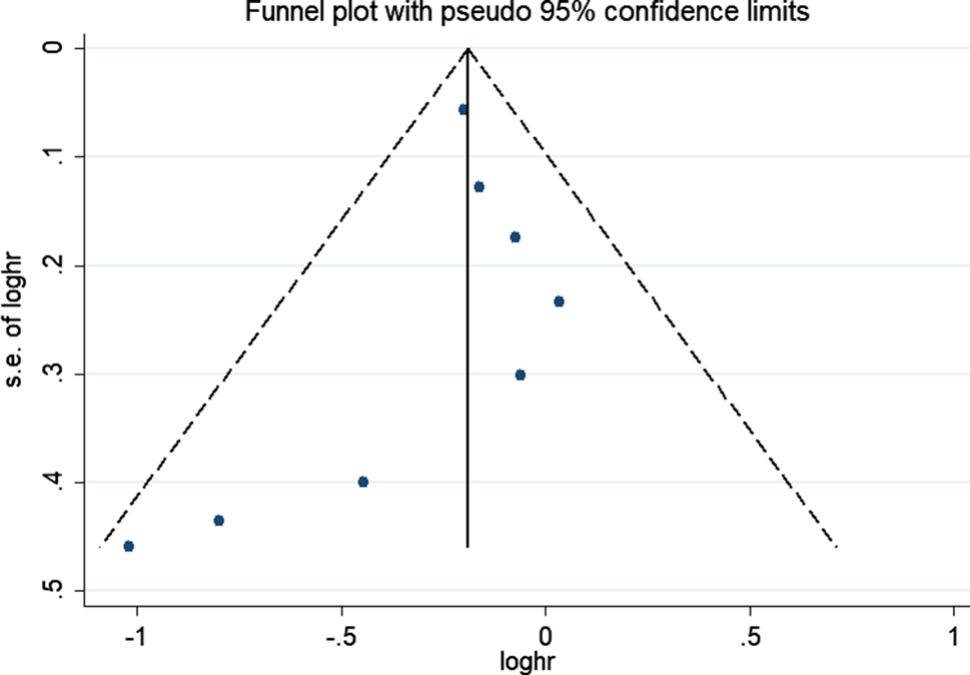
Funnel plot of the included studies

Figure 1 shows the summary profile of the search. We identified 11 potential cohort studies that examined peri-operative chemotherapy in combination with surgery for the treatment of resectable lung metastasis. Of the 11 studies, three studies were excluded because of insufficient data. Eight studies were eligible for the meta-analysis. Potential publication biases were evaluated using the funnel plot, which showed no publication bias (Fig. 2).

A total of 1936 patients in eight studies were evaluated in this meta-analysis, of whom 926 patients received surgery alone and 1010 patients received peri-operative chemotherapy and surgery (Guerrera et al. 2016, Park et al. 2016, Karim et al. 2017, Park et al. 2017, Shiomi et al. 2017, Hawkes et al. 2012, Kaira et al. 2011, Okumura et al. 2017). Characteristics of the included studies are summarized in Table 1. These studies were all published between 2011 and 2017. Three studies were conducted in Japan, two in Korea, one in Italy, one in Canada, and one in the United Kingdom. The sample size ranged from 51 to 785. Most studies evaluated the role of peri-operative chemotherapy after R0 (no residual disease) resection. Most patients received adjuvant chemotherapy, while others received neo-adjuvant chemotherapy or both. Chemotherapy regimens included oxaliplatin-based chemotherapy, irinotecan-based therapy, molecular targeted drugs, and 5-FU monotherapy. Chemotherapy regimen was not described in one study (Guerrera et al. 2016). All studies were retrospective analyses. The quality assessment of the included studies was evaluated by NOS. The scores ranged from 7 to 9. The characteristics of the included studies contained sex and age (Table S1).

**Table 1:** Characteristics of the included studies

Author (year)	Recruitment period	Region	Characteristic of patients	Chemotherapy	Chemotherapy regimen	Median follow-up (months)	5-year survival (100%)
Park (2016)	2002–2013	Korea	*N* = 221; Median age: 62 years (range 30–85 years); Male: 141; Female: 80;	Adjuvant	5-FU, TS-1, capecitabine, FOLFOX OR FOLFIRI	34.7	64.20%
Karim (2017)	2002–2009	Canada	*N* = 420; Median age: 64 years (range 17–87 years); Male: 251; Female: 169;	Adjuvant and neo-adjuvant	Oxaliplatin-based chemotherapy, Irinotecan-based therapy OR 5-FU monotherapy	NR	40%
Park (2017)	2001–2015	Korea	*N* = 91; Median age: 63 years (range 35–83 years); Male: 62; Female: 29;	Adjuvant	FOLFOX, FOLFIRI OR capecitabine	46	61.40%
Shiomi (2017)	2000–2014	Japan	*N* = 100; Median age: 64.7 years (± 10.0 years); Male: 56; Female: 44;	Adjuvant	Oxaliplatin-containing, Irinotecan-containing and molecular-target drugs	51	76%
Hawkes (2012)	1997–2009	UK	*N* = 51; Median age: 63 years (range 41–78 years); Male: 30; Female: 21;	Adjuvant and neo-adjuvant	Oxaliplatin, Irinotecan, Mitomycin, 5-FU, capecitabin alone OR molecular target drugs	60	72%
Kaira (2011)	2003–2008	Japan	*N* = 80; Median age: 66 years (range 33–81 years); Male: 53; Female: 27;	Adjuvant	FOLFOX OR uracil-tegafur/leucovorin	71	55.80%
Guerrera (2016)	2004–2012	Italy	*N* = 188; Median age: 66 years (range 58–72 years); Male: 109; Female: 79;	Adjuvant and neo-adjuvant	NR	45	53%
Okumura (2017)	2004–2008	Japan	*N* = 785; Median age: 66 years (range 29–89 years); Male: 448; Female: 337;	Adjuvant	Oxaliplatin-containin, Irinotecan-containing, 5-fluorouracil/leucovorin OR tegafur/uracil	65	68.10%

**Fig. 3:**
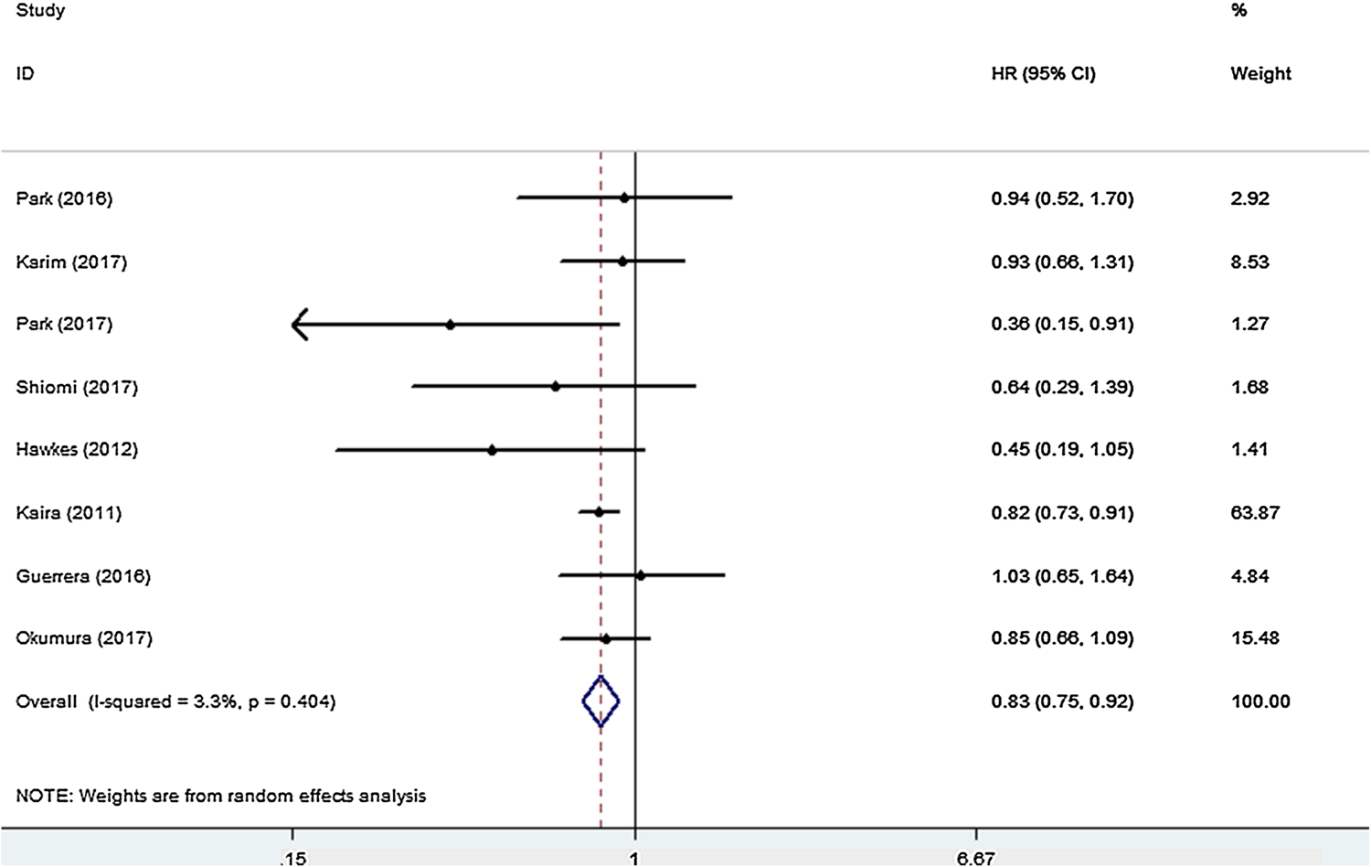
HR analysis of OS for peri-operative chemotherapy vs. surgery alone

**Fig. 4:**
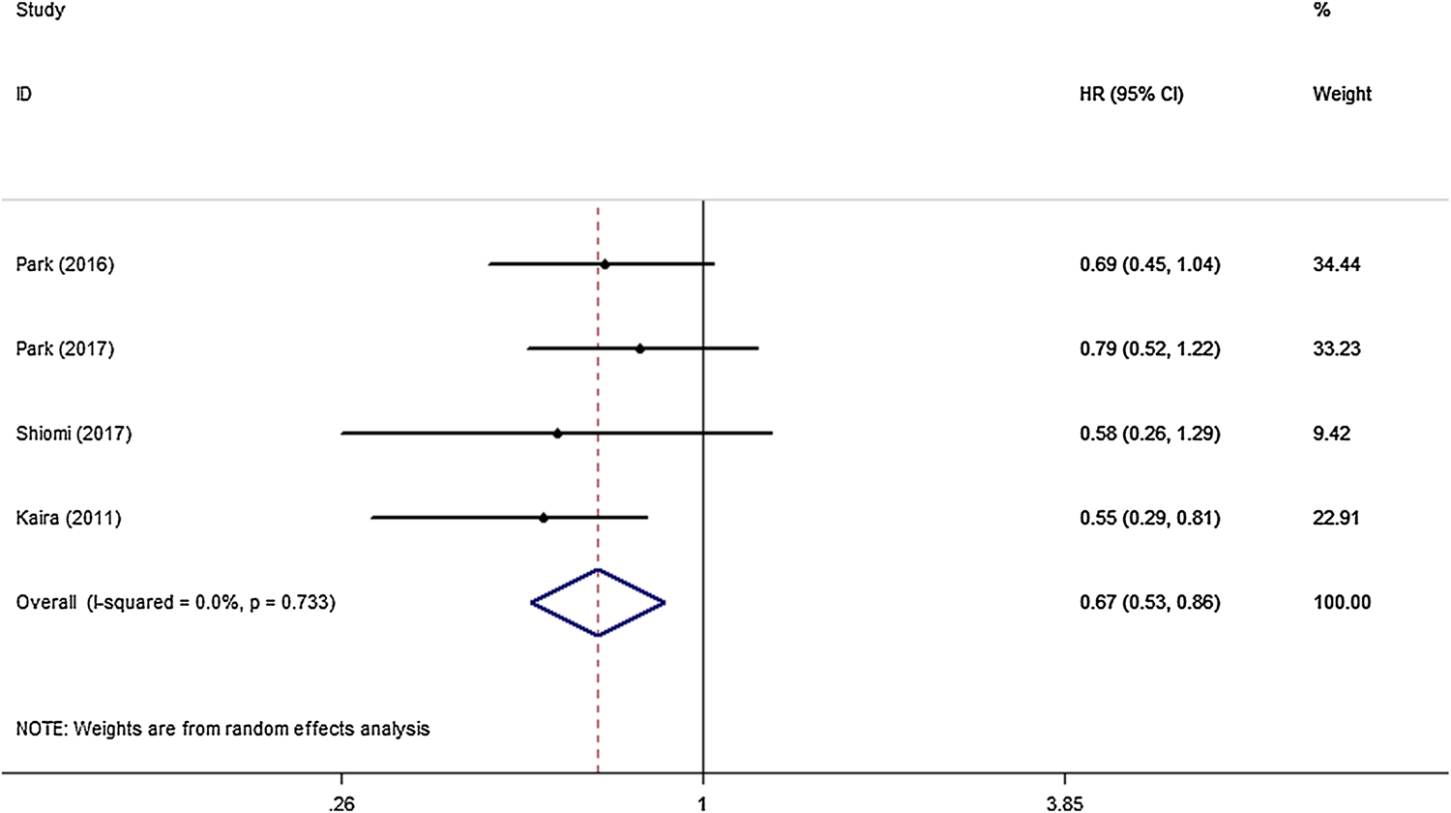
HR analysis of PFS/RFS/DFS for peri-operative chemotherapy vs. surgery alone

All eight studies that investigated OS were included for meta-analysis. The heterogeneity of the therapeutic effect was not significant (*p* = 0.404, *I*^*2*^ = 3.3%). The outcome of the random effect model of the meta-analysis revealed that treatment with peri-operative chemotherapy significantly prolonged OS in comparison to treatment with only pulmonary metastasectomy (HR 0.83, 95% CI 0.75–0.92, *p* < 0.05) (Fig. 3). PFS/RFS/DFS was described in four studies. In these four studies, chemotherapy was performed in the post-surgical setting. The meta-analysis results suggested that peri-operative chemotherapy reduced the risk of progression or recurrence (HR 0.67, 95% CI 0.53–0.86, *p* < 0.05) (Fig. 4). No heterogeneity was identified (*p* = 0.733, *I*^*2*^ = 0.0%). Independent prognostic factors were verified using multivariate analyses in four studies. Peri-operative chemotherapy strongly affected PFS/RFS/DFS and OS by multivariate analysis compared to surgery alone (OS: HR 0.56, 95% CI 0.36–0.86, *p* < 0.05; PFS/RFS/DFS: HR 0.64, 95% CI 0.46–0.87, *p* < 0.05) (Tables 2, 3). Different predictors were included in each final multivariate model, whereas all four studies adjusted for age, CEA level, tumor location, and peri-operative chemotherapy in the multivariate analysis.

**Table 2:** Subgroup analyses of HR of OS for peri-operative chemotherapy vs. surgery alone

Study of subgroup	Chemotherapy and surgery	Surgery alone	HR (95% CI)	Weight
Multivariate analysis				
Park (2017)	63	28	0.47 (0.11, 2.01)	8.88
Shiomi (2017)	42	58	0.35 (0.14, 0.81)	24.49
Guerrera (2016)	122	66	0.67 (0.40, 1.15)	66.62
Subtotal	227	152	0.56 (0.36, 0.86)	
Heterogeneity: *I*-squared = 0.0%, *p* = 0.444				
R0 resection				
Park (2016)	176	45	0.94 (0.52, 1.70)	19.23
Park (2017)	63	28	0.36 (0.15, 0.91)	9.82
Shiomi (2017)	42	58	0.64 (0.29, 1.39)	12.44
Hawkes (2012)	38	13	0.45 (0.19, 1.05)	10.76
Okumura (2017)	376	409	0.85 (0.66, 1.09)	47.74
Subtotal	695	553	0.72 (0.53, 0.97)	
Heterogeneity: *I*-squared = 0.0%, *p* = 0.425				
Metachronous pulmonary metastases				
Park (2017)	63	28	0.36 (0.15, 0.91)	47.35
Hawkes (2012)	38	13	0.45 (0.19, 1.05)	52.65
Subtotal	101	41	0.40 (0.22, 0.75)	
Heterogeneity: *I*-squared = 0.0%, *p* = 0.725

**Table 3:** Subgroup analyses of HR of PFS/RFS/DFS for peri-operative chemotherapy vs. surgery alone

Study of subgroup	Chemotherapy and surgery	Surgery alone	HR (95% CI)	Weight
Multivariate analysis				
Park (2016)	176	45	0.65 (0.42, 1.00)	53.95
Park (2017)	63	28	0.92 (0.43, 1.98)	17.16
Shiomi (2017)	42	58	0.49 (0.27, 0.88)	28.89
Subtotal	281	131	0.64 (0.46, 0.87)	
Heterogeneity: *I*-squared = 0.0%, *p* = 0.438				
R0 resection				
Park (2016)	176	45	0.69 (0.45, 1.04)	44.67
Park (2017)	63	28	1.79 (0.52, 1.22)	43.11
Shiomi (2017)	42	58	0.58 (0.26, 1.29)	12.22
Subtotal	281	131	0.72 (0.54, 0.95)	
Heterogeneity: *I*-squared = 0.0%, *p* = 0.779)				
Metachronous pulmonary metastases				
Park (2016)	151	41	0.57 (0.38, 0.87)	51.26
Park (2017)	63	28	0.79 (0.52, 1.22)	48.74
Subtotal	214	69	0.67 (0.49, 0.92)	
Heterogeneity: *I*-squared = 13.7%, *p* = 0.282				

The efficacy of peri-operative chemotherapy after pulmonary metastasectomy was further investigated in CRC patients after R0 resection. Five studies were included in the final analysis. Hawkes et al., and Okumura et al., did not report PFS/RFS/DFS data. There was a significant different benefit in the peri-operative group for OS and PFS/RFS/DFS (OS: HR 0.82, 95% CI 0.74–0.90, *p* < 0.05; PFS/RFS/DFS: HR 0.72, 95% CI 0.54–0.95, *p* < 0.05) (Tables 2, 3). Three of these studies assessed OS and PFS of CRC patients with metachronous lung metastases. Hawkes et al. provided data on OS, while Park et al. supplied data on PFS/RFS/DFS. A significant improvement was confirmed in OS and PFS/RFS/DFS (OS: HR 0.40, 95% CI 0.22–0.75, *p* < 0.05; PFS/RFS/DFS: HR 0.67, 95% CI 0.49–0.92, *p* < 0.05) (Tables 2, 3).

## Discussion

The need for peri-operative chemotherapy after pulmonary metastasectomy from CRC patients is still a matter of debate due to the lack of evidence. In this study, we evaluated eight retrospective studies concerning the efficacy of chemotherapy in these patients. The HR estimates suggested that patients benefit from peri-operative chemotherapy after resection of pulmonary metastases. For PFS/RFS/DFS, the peri-operative treatment group also improved survival. Multivariate meta-analyses were conducted to adjust for other variables that could have influenced survival, such as age, disease stage, CEA level, and tumor location. Our analyses demonstrated that peri-operative chemotherapy was an independent and favorable prognostic factor for OS and PFS/RFS/DFS, which is consistent with previous findings (Muñoz Llarena et al. 2007; Guerrera et al. 2017).

Surgery has become the standard treatment for CRC patients after resection of pulmonary metastases (Goya et al. 1989; Mitry et al. 2010; Zampino et al. 2014; Van Cutsem et al. 2016). Despite the recommendation of adjuvant chemotherapy after surgery for CRC patients with liver metastases (Nordlinger et al. 2008, Van Cutsem et al. 2016), this is not the case for CRC patients with lung metastases (Brandi et al. 2016; Guerrera et al. 2017). “Watch and wait” is regarded to be an appropriate approach after pulmonary metastasectomy in CRC patients for lung involvement, as it may be associated with a better outcome. The different therapeutic recommendation may be due to molecular differences depending on the sites of metastases (Khattak et al. 2012; Yaeger et al. 2014). However, our results showed that peri-operative chemotherapy led to a significant OS and PFS/RFS/DFS benefit in CRC patients in comparison to surgery alone. We further analyzed studies that investigated R0 resection of pulmonary metastases or metachronous pulmonary metastases. Survival benefits in terms of OS and PFS were also achieved by peri-operative chemotherapy. The benefit of peri-operative chemotherapy may be influenced by the type of chemotherapy regimen received by CRC patients (Nakajima et al. 2017).

There were some limitations to this study. All included studies were retrospective analyses and were associated with a low level of evidence. Compared with randomized control trials, retrospective and non-randomized studies can introduce bias into data analysis and confound the results. For example, adjuvant chemotherapy was likely proposed to a selected subgroup of patients who were fit and without serious complications after surgery, had poor prognosis, or had heavier disease burden. It is also necessary to consider the heterogeneous chemotherapy regimens used in the study. Chemotherapy regimens used in the study included intravenous 5-FU, TS-1, capecitabine, intravenous 5-FU plus oxaliplatin (FOLFOX), intravenous 5-FU plus irinotecan (FOLFIRI), and molecular targeted agents. Recently, these new chemotherapeutic regimens have significantly improved the prognosis of patients with CRC (de Gramont et al. 2000; Saltz et al. 2001; Goldberg et al. 2004). The survival rates of patients treated with chemotherapy after resection of CRC pulmonary metastases have increased over time. This has contributed to the wide range in the efficacy of peri-operative chemotherapy in CRC patients who underwent pulmonary metastectomy. However, it is still difficult to define the chemotherapy regimens, doses, and the number of cycles of peri-operative therapy in practice.

## Conclusion

In conclusion, our study is the first meta-analysis to assess whether peri-operative chemotherapy impacts the survival of CRC patients with resectable pulmonary metastases. Although a significant survival benefit has been observed in these patients, the conclusion is still limited by the fact that the analysis was based on retrospective studies. Randomized control studies focused on peri-operative treatment for this subgroup of patients is warranted.

## Electronic supplementary material

Below is the link to the electronic supplementary material.

Supplementary table 1 (XLS 20 kb)

## Abbreviations

CRC: Colorectal cancer
HR: Hazard ratio
OS: Overall survival
PFS: Progression-free survival
RFS: Recurrence-free survival
DFS: Disease-free survival
CEA: Carcinoembryonic antigen
DFI: Disease-free interval
NOS: Newcastle–Ottawa Scale
FOLFOX: Intravenous 5-FU plus oxaliplatin
FOLFIRI: Intravenous 5-FU plus irinotecan
